# Toward a User-Accessible Spectroscopic Sensing Platform for Beverage Recognition Through K-Nearest Neighbors Algorithm

**DOI:** 10.3390/s25144264

**Published:** 2025-07-09

**Authors:** Luca Montaina, Elena Palmieri, Ivano Lucarini, Luca Maiolo, Francesco Maita

**Affiliations:** National Research Council (CNR), Institute for Microelectronics and Microsystems (IMM), 00133 Rome, Italy; luca.montaina@artov.imm.cnr.it (L.M.); elena.palmieri@artov.imm.cnr.it (E.P.); ivano.lucarini@cnr.it (I.L.); francesco.maita@cnr.it (F.M.)

**Keywords:** sensing platform, diet monitoring, beverage recognition, public health, machine learning, KNN, IoT devices, smart cutlery

## Abstract

Proper nutrition is a fundamental aspect to maintaining overall health and well-being, influencing both physical and social aspects of human life; an unbalanced or inadequate diet can lead to various nutritional deficiencies and chronic health conditions. In today’s fast-paced world, monitoring nutritional intake has become increasingly important, particularly for those with specific dietary needs. While smartphone-based applications using image recognition have simplified food tracking, they still rely heavily on user interaction and raise concerns about practicality and privacy. To address these limitations, this paper proposes a novel, compact spectroscopic sensing platform for automatic beverage recognition. The system utilizes the AS7265x commercial sensor to capture the spectral signature of beverages, combined with a K-Nearest Neighbors (KNN) machine learning algorithm for classification. The approach is designed for integration into everyday objects, such as smart glasses or cups, offering a noninvasive and user-friendly alternative to manual tracking. Through optimization of both the sensor configuration and KNN parameters, we identified a reduced set of four wavelengths that achieves over 96% classification accuracy across a diverse range of common beverages. This demonstrates the potential for embedding accurate, low-power, and cost-efficient sensors into Internet of Things (IoT) devices for real-time nutritional monitoring, reducing the need for user input while enhancing accessibility and usability.

## 1. Introduction

Food plays an essential and irreplaceable role in human life, serving not only as a source of nourishment and energy but also as a key factor in the social sphere [1]. A healthy diet can indeed positively impact our health, sleep [2,3], behavior [4], and overall well-being [5,6,7,8], leading us to experience a healthier and longer life. On the other hand, studies suggest that an unhealthy diet poses a significant risk factor for the development of chronic diseases like metabolic disorders or cardiovascular diseases [9,10].

Nowadays, the fact that highly processed and calorie-dense foods are not only widely available, but also often inexpensive, has increased concern about the food we daily consume. Indeed, the abundance and easy availability of unhealthy food options contributes significantly to the increasing rates of obesity and related health issues, such as heart diseases, diabetes, and other chronic conditions [10,11,12]. These factors, combined with modern lifestyles, are leading to the fact that obesity and related diseases are increasing globally [13,14,15].

For these reasons, carefully monitoring what we eat and drink is a crucial aspect of maintaining a healthy lifestyle, particularly for individuals adhering to specific dietary plans or managing health conditions. By being mindful of our food and beverage choices, we can ensure that we meet our nutritional needs, support overall wellness, and prevent potential health risks. Whether it is for weight management, controlling chronic illnesses, or optimizing fitness, paying close attention to our diet allows us to make informed decisions that align with our personal health goals and long-term well-being [16].

Nowadays, technological advancements are driving the development of increasingly non-invasive devices that help monitor various parameters, including fitness levels [17], vital signs [18,19], and nutritional intake. Indeed, the traditional method of recording nutrient intake involves manually tracking food consumption to monitor calories, macronutrients, and other dietary components. However, while effective, this method is often time-consuming and labor-intensive, requiring consistent effort and attention by the user [20,21]. As a result, many people find it challenging to maintain such an approach long term, highlighting the need for more efficient tools and technologies to simplify dietary monitoring. For this reason, there has been a growing surge in the development of mobile apps designed to help individuals recognize and track their food intake more easily. Many of these apps leverage the camera systems of smartphones, allowing users to simply take pictures of their meals for automated analysis. These programs exploit artificial intelligence and machine learning to identify food items, estimate portion sizes, and provide detailed nutritional information, making it easier to monitor calorie and nutrient intake. This innovation significantly reduces the time and effort required for manual tracking, offering a more convenient solution for maintaining a healthy diet [22,23,24,25].

While this approach greatly simplifies the process of gathering nutritional information, it still relies on a certain level of smartphone knowledge and requires users to take a picture of each dish and beverage consumed. This reliance on smartphone use can be challenging for two reasons. First, it can be difficult or even unfeasible for individuals who lack familiarity with or access to smartphone technology, such as older adults or people with certain disabilities. Second, even for tech-savvy users, smartphone apps may struggle to distinguish between visually similar foods or beverages. For instance, identifying subtle variations in dishes with similar ingredients can lead to inaccuracies in tracking. In addition, having these apps access smartphone cameras also raises privacy issues about the use and collection of personal data derived from the uploaded images. These limitations underscore the need for further technological advancements and alternative tracking solutions that can accommodate a broader range of users while improving the accuracy of food recognition tools. In this context, we propose a compact tool that can be implemented in a smart glass or smart cup specifically designed to distinguish among the most common beverages. This system utilizes a commercially available spectroscopy sensor, the AS7265x (AMS Osram, Premstaetten, Austria), to detect the unique spectral properties of each beverage. The classification of each beverage is enabled by a machine learning model that applies the K-nearest neighbor (KNN) algorithm. We apply the KNN algorithm to compare the spectral data of an unknown beverage with the stored data of known beverages, matching it to the closest categories based on their spectral “fingerprints”. This innovative approach opens to a reliable and easy-to-use solution for beverage recognition. In the contest of IoT technology development, these types of systems enable the design of intelligent food monitoring solutions, such as smart plates, cups or utensils equipped with spectroscopic sensors, and machine learning algorithms that can autonomously analyze the composition of food and beverages in real time, providing users with detailed nutritional information without requiring manual input.

## 2. Materials and Methods

### 2.1. Beverages Analyzed

The beverages selected for testing were chosen to represent a wide range of popular options, covering diverse categories to ensure the system’s versatility. The selected beverages reported in Table 1 included both animal and plant-based milks, still and sparkling water, various sports and energy drinks, sugary beverages, wine, as well as commonly consumed drinks like tea and coffee. By including such a broad spectrum, we aimed to capture the unique spectral profiles of each beverage type, thereby enhancing the system’s capability to accurately differentiate among them. Although different beverages can be consumed at different temperatures, in this study we decided to analyze the beverage at room temperature to ensure consistency and excluding the temperature effects on the beverage spectrum (a simple example of the temperature on the spectra is reported in Figure S1). Thirty spectral measurements were taken for each drink without any dilution, allowing us to capture a comprehensive spectral profile, reducing the influence of potential measurement noise or variability. Each beverage has been tested filling a commercial 4.5 mL cuvette (10 mm optical path) by Kartell LABWARE (Noviglio (MI), Italia). Poly(methyl methacrylate) (PMMA) was chosen as the material for the cuvette to facilitate the design, prototyping and development of an intelligent IoT cup. In fact, its suitability with precision machining or molding makes it ideal for fabricating integrated optical paths with controlled geometry. Moreover, its compatibility with embedding electronic components within the container walls supports the development of sealed and washable smart devices, where sensor alignment and measurement stability can be ensured by design. The cuvette was washed after each measurement and replaced between different beverages.

### 2.2. System Setup

**Figure 1:**
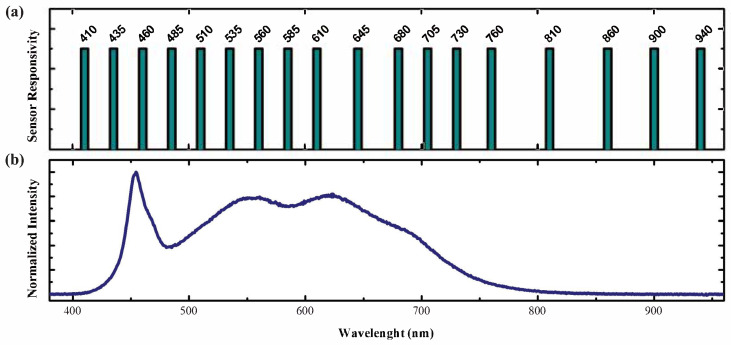
(**a**) Distribution of peak absorption for the AS7265x kit sensor [26] and (**b**) normalized emission spectra of the LED as measured with Photonic Multi-channel Analyzer PMA-12.

**Figure 2:**
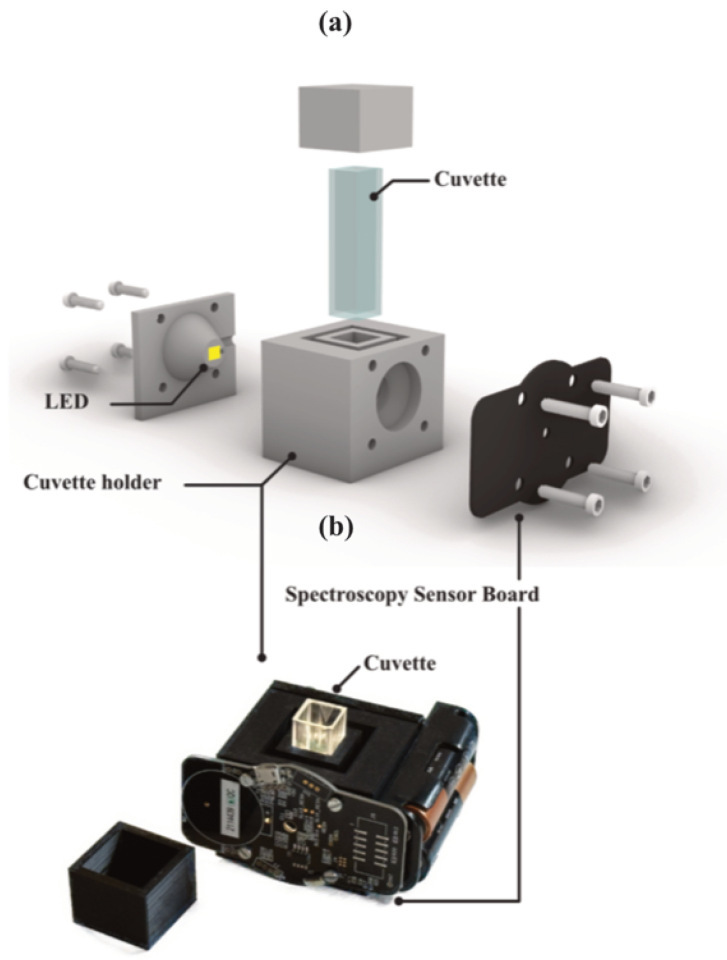
(**a**) 3D renders and (**b**) photo of the system setup.

The sample spectrum is processed by an AS7265x kit, a spectroscopic sensor with integrated interference filters deposited on CMOS silicon. The AS7265x kit from Ams integrated three sensors, each with six independent optical channels. In Figure 1a is reported the spectral response of the 18 channels of the Ams kit [26]: the three sensors thus offer a combined spectroscopic range from 410 to 940 nm with each channel providing a full width at a half maximum of 20 nm. The system uses a 4000 K white LED from Cree LED Inc. (Durham, NC, USA) as its light source. Its emission was acquired through a Photonic Multi-channel Analyzer PMA-12 detector by Hamamatsu Photonics (Hamamatsu City, Shizuoka, Japan) and is reported in Figure 1b. This LED was selected due to the Color Rendering Index (CRI) of 90, ensuring a wide spectral power distribution that provides an improved spectral illumination compared to a standard white LED. The 3 V supply power needed for the illumination source was provided through batteries, which ensured the operation of the LED during the entire data acquisition. The absorption spectra were acquired using the software Spectral Sensor Dashboard (v.5.1.0) from Ams. A 3D-printed model provided a structure to mount the components. The complete system setup is reported in Figure 2.

### 2.3. KNN Algorithm

**Figure 3:**
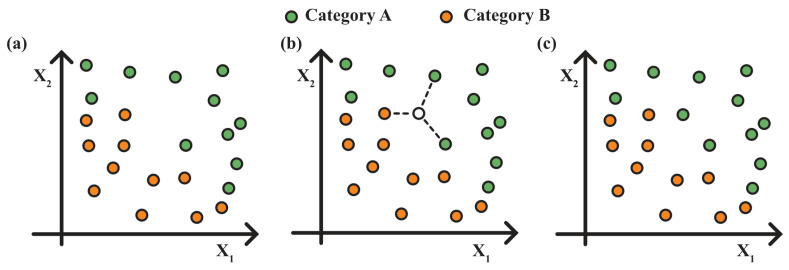
Two-dimensional example of KNN (K = 3) classification algorithm: (**a**) training dataset with two classes (labeled green and orange dots). (**b**) A new unclassified data is added to the graph (white dot) and compared with the 3 nearest neighbors. (**c**) The new data is then assigned to the category whose number of first neighbors is highest (green).

One of the most simple and effective models to predict the discrete class label of an unlabeled sample is the KNN algorithm. KNN is a non-parametric supervised machine learning algorithm often used for data classification [27]. KNN algorithm operates on the principle of proximity. When presented with a new data point, the algorithm calculates the distance between the new data point and all points in the training dataset. Based on the calculated distances, it selects the K nearest data points from the training dataset. Finally, for classification task, the algorithm assigns the new data point to the majority class among its K neighbors [27,28,29,30]. An example of KNN is represented in Figure 3.

To evaluate the performance of the model, the data have been divided into two sets: training data and test data. The training data are used to teach the algorithm the unique characteristics and features of each class, enabling it to build a classification model. The test data are used to assess the model’s performance. This evaluation involves comparing the model’s predictions with the actual classifications, thereby determining its accuracy, and overall effectiveness. This approach ensures that the model is not only well trained but also capable of generalizing its classification ability to new, unseen data. This aspect is particularly relevant for this application, the number and typology of beverages being very large. In particular, we use a k-fold cross-validation, which divides the dataset into k_f_ parts, training the model on k_f_—1 fold and testing on the remaining fold, repeating the process k_f_ times. By using k-fold cross-validation, a more reliable estimation of the model can be obtained because each observation in the dataset is used for both training and testing. To have a good balance between computational efficiency and reliable model evaluation, we chose a k_f_ = 3 [31,32,33,34,35]. In the case of smart IoT cup development, the sensor acquires the absorption spectrum of a beverage, obtaining the characteristic components of the spectrum. These are then compared with a database of labeled beverage spectra using the Euclidean distance and the KNN algorithm identifies the closest K spectra from the training set. The beverage is then classified according to the majority class among these neighbors.

The performance of the KNN algorithm depends significantly on the choice of K number of nearest neighbor. For this reason, we systematically tested all values of K ranging from 1 to 15 during the test of the sensor. This allowed us to determine the K value that maximizes the classification.

The KNN algorithm applied in this study was implemented using the open-source scikit-learn Python library (v.1.7).

## 3. Results and Discussion

**Figure 4:**
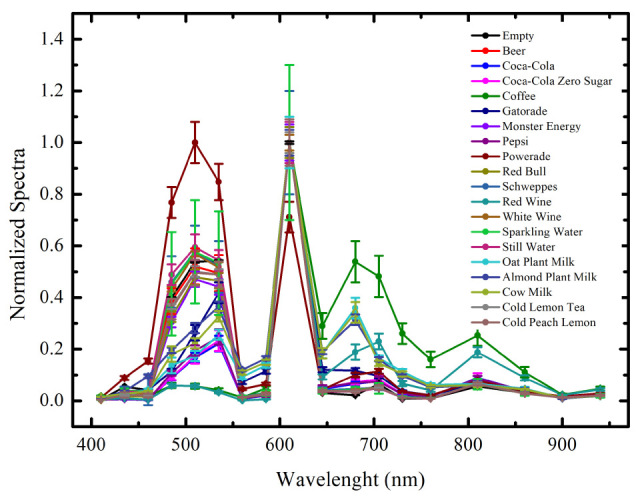
Normalized beverage light absorption as a function of the wavelength.

Figure 4 shows the intensities of light absorbed by the beverages, normalized to their respective maximum values, plotted as a function of the sensor wavelengths. As can be seen from the absorption graph, most of the spectra of the beverages vary significantly in intensity at specific wavelengths. These variations form the basis for distinguishing between different drinks using the KNN algorithm. The spectra of some drinks, on the other hand, are found to have small differences between the values of absorbed light intensities, resulting in a spectral fingerprint that is not easily distinguishable. Very similar spectra, such as those found from Coca-Cola^®^ and Coca-Cola Zero Sugar^®^, may therefore result in a less accurate classification.

**Figure 5:**
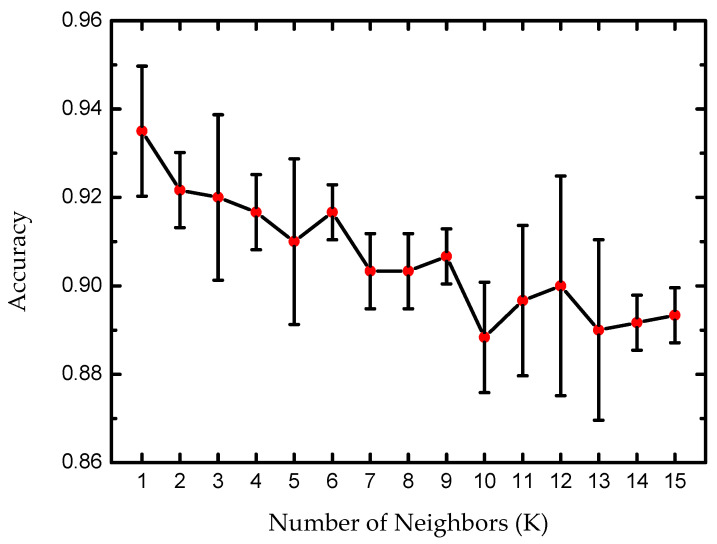
Accuracy of the KNN model as a function of the K nearest neighbors as average (red dots) respect to the several K-fold tests performed.

Data analysis began with a study of the number of K nearest neighbors that would maximize the accuracy of the classification model employed. In Figure 5 is reported the accuracy of the model as a function of the number of neighbors (K) used in the K-nearest neighbors’ algorithm. The k-fold cross-validation revealed that the model achieves the highest accuracy (around 93%) when K is set to smaller values, specifically at K = 1. As K increases, the accuracy gradually declines. This suggests that a smaller neighborhood size allows the model to make more precise classifications, where the distinctions between some beverage classes are sharp.

**Figure 6:**
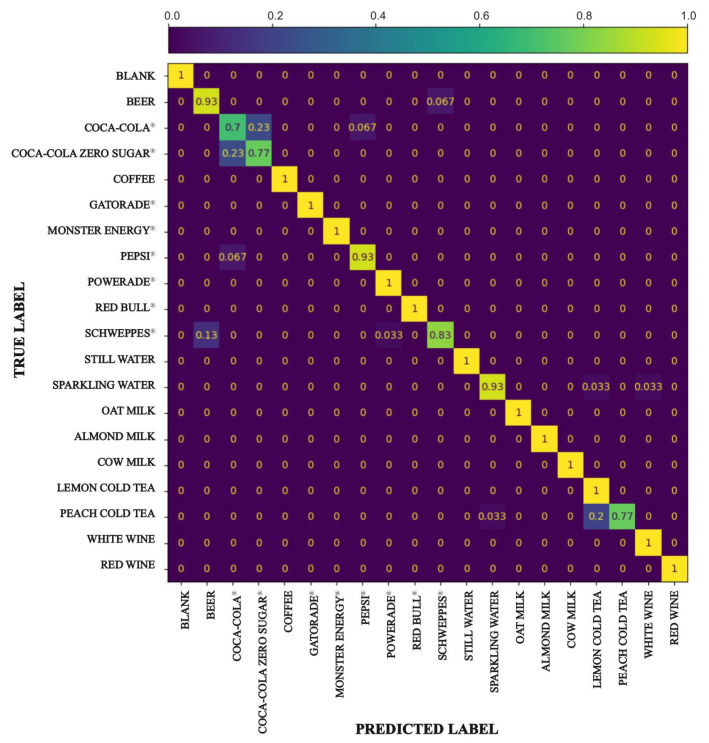
Confusion matrix of the KNN model (K = 1), showing the classification performance across the different beverages.

To obtain a more detailed description of the performance of the KNN classifier, we produced a confusion matrix for K = 1 (Figure 6). Analysis of the confusion matrix reveals a good accuracy of the KNN model used. In fact, as can be seen from the diagonal of the matrix, most drinks are recognized with 100% accuracy. This suggests that the spectral profiles of most of the beverages studied are distinctive and the model correctly succeeds in classifying them.

Other beverages, however, are recognized with more difficulty by the model. In fact, beverages that have a spectrum with few differences are classified with lower accuracy.

It is worth observing how beverages such as still and sparkling water are well distinguished from the model. The presence of dissolved CO_2_ in sparkling water likely plays a key role in differentiating these two beverages. CO_2_ not only acidifies the water, potentially altering the solubility of various substances, but also generates bubbles that induce light scattering within the cuvette. This scattering is expected to modify the intensity profile detected by the sensor [36], providing distinguishable features between the still and the sparkling water, and, at the same time, creating confusion in the classification of other beverages. In particular, two beverages show noticeable confusion: Coca-Cola^®^ being misclassified as Coca-Cola Zero Sugar^®^ and vice versa. This is likely because their visible spectral profiles are very similar due to similar ingredients and composition.

**Figure 7:**
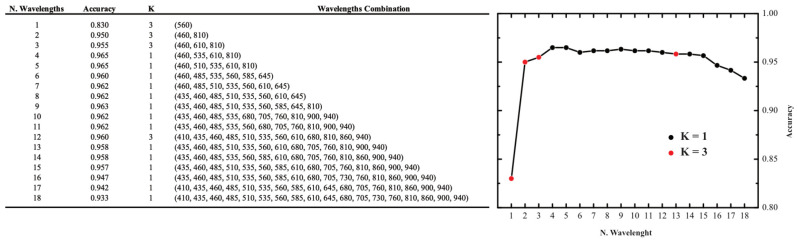
Accuracy of the KNN model as a function of the wavelengths combination.

Subsequently, we focused on optimizing the classification process while reducing the number of sensors required for measurement. In fact, using a smaller number of wavelengths can significantly reduce the costs associated with developing an IoT device while also lowering its power consumption. Additionally, analyzing fewer wavelengths decreases computational complexity, making data processing more efficient. As a result, this approach could not only minimize production and operational costs but also extends the battery life of an IoT device. Therefore, our purpose is to verify which and how many wavelengths are necessary to distinguish the drinks. In fact, as can be seen from the analysis of Figure 4, with this setup, some wavelengths show a greater difference in intensity between beverages making them ideal for classification. In contrast, at other wavelengths where intensity measurements overlap, the beverages become indistinguishable. To address this, we developed an algorithm to systematically evaluate all possible combinations of wavelengths and identify the configuration that maximizes the accuracy of the KNN model. Specifically, we utilized the itertools.combinations module to generate every possible combination of wavelengths, ranging from one wavelength configuration to those including all 18 available wavelengths. Using the same threefold cross-validation (k_f_ = 3) employed in the previous model, we calculated the classification accuracy as a function of the number of wavelengths included in the model. This approach allowed us to identify the minimal set of wavelengths that preserved or enhanced classification accuracy while minimizing the complexity of the measurement system. In Figure 7 are reported the obtained results.

The table reveals an interesting trend regarding the impact of the number of sensors on model accuracy. Contrary to intuition, using all 18 sensors does not result in the highest accuracy for the KNN model. Instead, the accuracy improves as the number of sensors increases up to four or five sensors, after which it gradually declines. The best performance, combining high accuracy with the minimum number of optical channels, is achieved using four sensors. Specifically, selecting wavelengths at 460, 535, 610, and 810 nm yields an accuracy of 96.5% when K = 1. The fact that the best accuracy is not obtained with the maximum number of wavelengths indicates that not all channels contribute positively to the KNN model. Optical channels where the absorbed light intensity does not exhibit clear distinctions between beverage classes can introduce noise or ambiguity, leading to model confusion. This reduces classification accuracy by blurring the boundaries between different beverage categories.

**Figure 8:**
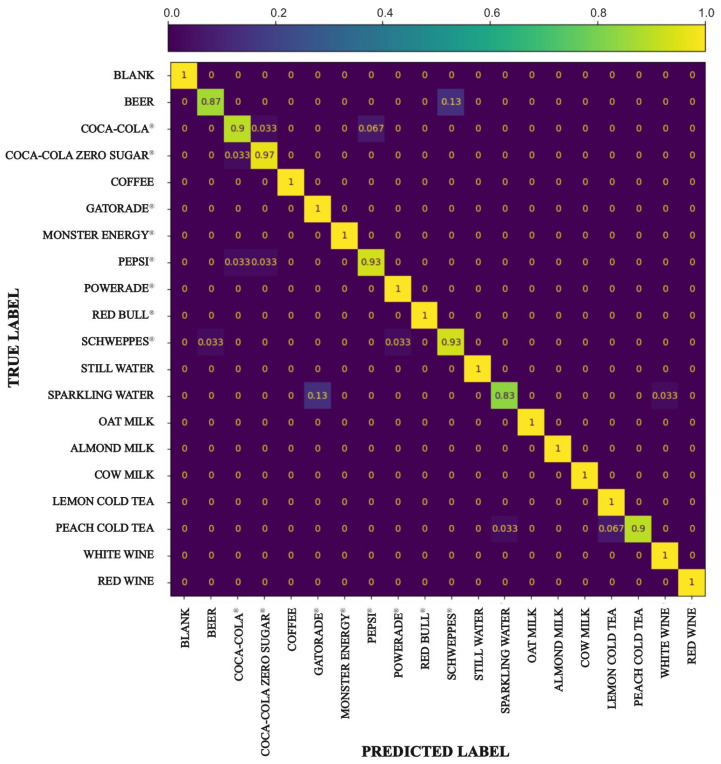
Confusion matrix of the KNN model (K = 1), illustrating beverage classification performance using the optimized wavelengths (460, 535, 610, and 810 nm).

After optimizing the use of sensors, we recalculated the confusion matrix, this time considering only the data corresponding to the four wavelengths (460, 535, 610, and 810 nm) that were identified as maximizing the model’s accuracy (Figure 8). By restricting the analysis to these selected wavelengths, we aimed to assess the effectiveness of the reduced dataset in maintaining high classification accuracy across all beverage classes.

A comparison with the confusion matrix generated using the full set of wavelengths (Figure 6) reveals some differences in performance when we consider only the optimized subset of wavelengths. While there is a general improvement in the model’s overall accuracy, this improvement is not evenly distributed across all the beverages tested. Specifically, the model shows enhanced ability to identify beverages such as Coca-Cola^®^, Coca-Cola Zero Sugar^®^, Schweppes^®^, Pepsi^®^, and cold peach tea, which now demonstrate clearer separation and reduced misclassification.

However, this improvement comes with certain trade-offs. Beverages such as beer and sparkling water are recognized with greater difficulty by the algorithm when using only the optimized wavelengths. This suggests that the model relies on information provided by the wavelengths excluded during optimization to distinguish these beverages.

## 4. Conclusions

In this paper, we introduced a novel approach for automatic beverage recognition, leveraging the combination of spectroscopic analysis and machine learning techniques. A spectrum consisting of 18 discrete wavelength points was captured using a commercial optical sensor, and the data was analyzed using a KNN machine learning model, achieving over 96% accuracy in distinguishing among a variety of commonly consumed beverages.

Moreover, this research paves the way for the development of compact, cost-effective, and automated mobile sensors capable of recognizing beverages in real time in smart cutlery tools like smart cups. Such devices could enable individuals to effortlessly monitor their nutritional drink intake, promoting healthier lifestyles and facilitating adherence to dietary plans. Future developments may extend this approach to include broader beverage categories, refine classification for challenging cases, and integrate additional sensor types or preprocessing methods to enhance robustness and reliability.

## Figures and Tables

**Table 1:** List of beverages chosen to test the model.

Beverage Category	Beverage Type
Water	Still Water, Sparkling Water
Animal based Milk	Cow Milk
Plant based Milk	Oats Milk, Almond Milk
Soft Drink	Coca-Cola^®^, Coca-Cola Zero Sugar^®^, Pepsi^®^,
	Schweppes^®^, Lemon Cold Tea, Peach Cold Tea
Alcoholic Drink	Beer, White Wine, Red Wine
Energy Drink	Monster Energy^®^, Red bull^®^
Sport Drink	Gatorade^®^, Powerade^®^
Brewed Drink	Coffee

## Data Availability

Data presented in this study are openly available upon request.

## Supplementary Materials

The following supporting information can be downloaded at: https://www.mdpi.com/article/10.3390/s25144264/s1. Supplementary Figure S1. (a) Normalized beverage light absorption and (b) confusion matrix of the KNN model.

## Abbreviations

The following abbreviations are used in this manuscript:

IoT	Internet of Things
CRI	Color Rendering Index
KNN	K-nearest neighbors
PMMA	Poly(methyl methacrylate)
