# Supplementary material for: Toward a User-Accessible Spectroscopic Sensing Platform for Beverage Recognition Through K-Nearest Neighbors Algorithm
Source: Sensors (Basel). 2025 Jul 9;25(14):4264. doi: 10.3390/s25144264 (PMC12299580; doi:10.3390/s25144264)
Supplement: Supplementary file 1 [file sensors-25-04264-s001.zip › Supplementary Material.pdf]

Supplementary Material

Toward a user-accessible spectroscopic sensing platform for beverage recognition through K-Nearest Neighbors Algorithm

Luca Montaina, Elena Palmieri, Ivano Lucarini, Luca Maiolo* and Francesco Maita

National Research Council (CNR) - Institute for microelectronics and microsystems (IMM), Rome, Italy; luca.montaina@artov.imm.cnr.it (L.Mo.); elena.palmieri@artov.imm.cnr.it (E.P.); ivano.lucarini@cnr.it (I.L.); francesco.maita@cnr.it (F.M.)
* Correspondence: luca.maiolo@cnr.it (L.Ma.)

Among the various factors that may influence the spectroscopic response of beverages, temperature is undoubtedly one of the most critical. Beverages are typically consumed in a wide range of temperatures, and given the small volume of liquid contained in a cup or glass, rapid temperature changes are likely to occur during real-world use. These variations can potentially alter the optical properties of the samples and, consequently, affect the sensor readings.
To assess whether the sensor is sensitive to temperature variations, we conducted a preliminary experiment using coffee samples at three different temperatures: 10 ± 3 °C, 30 ± 1 °C, and 60 ± 3 °C. Temperatures were monitored with a digital thermometer to ensure consistency across measurements. For each temperature condition, 30 spectral acquisitions were performed using the same experimental setup described in the main text. The acquired spectra were then classified using the K-nearest neighbors (KNN) algorithm, and the optimal value of K for this dataset was found to be 2. The results showed that the model successfully distinguished the coffee samples at 3 different temperatures, demonstrating that temperature can influence spectral features and may need to be considered in future smart cup applications. While this observation lies beyond the scope of the current work - which focuses primarily on demonstrating the feasibility of beverage classification using low-cost, easy to embed electronics - it highlights an important consideration for future smart cup applications. Accordingly, we opted to perform all measurements under controlled room-temperature conditions to preserve consistency and repeatability.

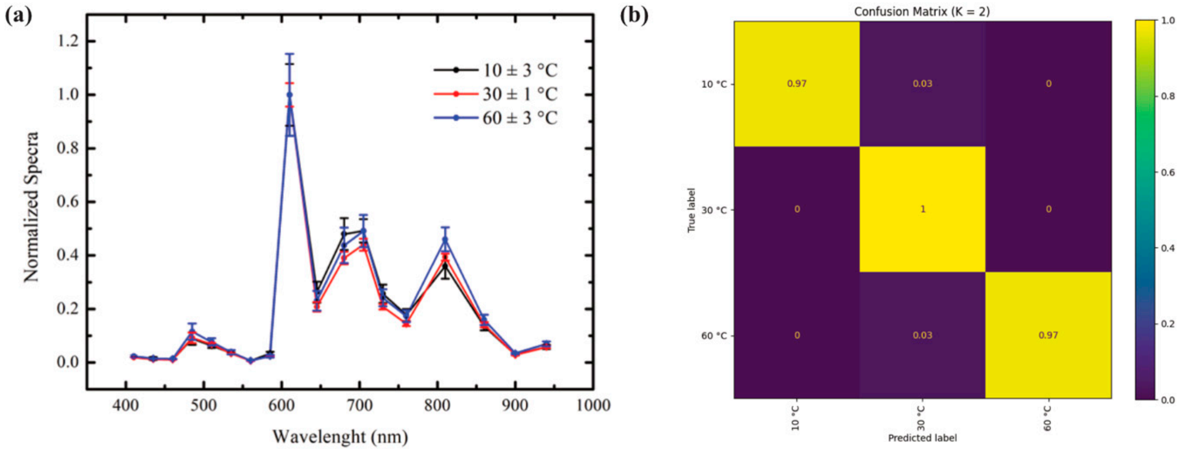

Figure S1: (a) Normalized beverage light absorption and (b) confusion matrix of the KNN model for the three different temperatures of the coffee samples.
